# Changes in Serum Creatinine May Cause Hypoglycemia among Non-Critically Ill Patients Admitted to Internal Medicine Units

**DOI:** 10.3390/jcm11226852

**Published:** 2022-11-20

**Authors:** Boris Zingerman, Israel Khanimov, Mordechai Shimonov, Mona Boaz, Benaya Rozen-Zvi, Eyal Leibovitz

**Affiliations:** 1 Department of Nephrology at The Hasharon Campus, Rabin Medical Center, Petah Tikva 49100, Israel; 2 Sackler Faculty of Medicine, Tel Aviv University, Tel Aviv 69978, Israel; 3 Department of Surgery “A”, Edith Wolfson Medical Center, Holon 58100, Israel; 4 Department of Nutrition Sciences, Ariel University, Ariel 40700, Israel; 5 Laniado Sanz Medical Center, Department of Internal Medicine “B”, Laniado Hospital, Netanya 42150, Israel; 6 Adelson School of Medicine, Ariel University, Ariel 40700, Israel

**Keywords:** creatinine, acute kidney injury, hypoglycemia, diabetes mellitus

## Abstract

Background: The association between changes in serum creatinine levels and hypoglycemia during hospitalization was investigated. Methods: This was a retrospective analysis of medical charts. Patients were categorized as having significant change in creatinine (SCIC) when serum creatinine levels rose or dropped ≥ 0.3 mg/dL from admission values at any time during their hospitalization. Patients were considered hypoglycemic if they had at least one documented glucose level ≤ 70 mg/dL during the hospitalization. Multiple logistic, linear and Cox regression analyses were used to ascertain the association between incident SCIC, severity and timing with incident hypoglycemia. Results: Included were 25,400 (mean age 69.9 ± 18.0, 49.3% were males). The rate of SCIC was 22.2%, and 62.2% of them were diagnosed upon admission. Patients with SCIC had a higher incidence of hypoglycemia compared to patients without (13.1% vs. 4.1%, respectively, *p* < 0.001). Patients with SCIC had an increased risk of hypoglycemia (OR 1.853, 95% CI 1.586–2.166, *p* < 0.001). The magnitude of SCIC was associated with the incidence (OR 1.316, 95% CI 1.197–1.447, *p* < 0.001) and the number of events (HR 0.054, 95% CI 0.021–0.087, *p* = 0.001). More than 60% of patients with hypoglycemia had their first event documented during days 0–6 after SCIC occurrence. Of those, the majority of events occurred on day 0–1, and the rate showed a gradual decrease throughout the first 5 days from SCIC occurrence. The results were similar for patients with and without DM. Conclusions: Changes in creatinine during hospitalization may cause hypoglycemia among patients admitted to internal medicine departments, regardless of DM status.

## 1. Introduction

Glucose control, even for the short duration of the hospital stay, significantly affects prognosis regardless of the reason for admission, both in terms of the length of hospital stay and mortality [1,2]. Hypoglycemia was shown repeatedly to be a key factor associated with poor outcome and independently increase morbidity and hospital mortality among several patient population, regardless of diabetes mellitus (DM) status [3,4]. 

Kidneys play a significant role in glucose homeostasis [5], and renal failure was found to be associated with hypoglycemia [6,7,8]. Acute kidney injury (AKI) is a commonly encountered syndrome characterized by the rapid loss in kidney’s function. AKI is the manifestation of a variety of diseases that lead to an abrupt decrease in glomerular filtration. This syndrome is a significant cause of morbidity and mortality in both high- and low-income countries [9]. The incidence of AKI is about 13.3 million cases per year, and it is responsible for about 1.7 million deaths annually [10]. Moreover, AKI was found to be associated with an increased risk of death even after 10 years from occurrence [11]. At present, hardly any therapeutic approaches exist for the treatment of AKI, despite its global increase in incidence [12]. 

The association between AKI and hypoglycemia was not investigated. In the current study, our aim was to find out the association between significant change in creatinine (SCIC) levels and incident hypoglycemia among patients with and without diabetes mellitus (DM), admitted to internal medicine departments. 

## 2. Methods

*Informed Consent*: The study was approved by The Institutional Review Board (IRB) of Edith Wolfson Medical Center. This study is considered an observational study by the IRB of Edith Wolfson Medical Center, and a signed informed consent was waived. All research was performed in accordance with relevant guidelines/regulations. Confirmation number: 0218-10-WOMC.

*Study population and inclusion criteria*: Included were all patients admitted and discharged from Internal Medicine units between 1 January 2015 and 31 December 2018 at a tertiary hospital in the center in the district of Tel Aviv, Israel. All patients were admitted to the units from the emergency department. For patients with multiple admissions, only the first was included. We excluded patients with creatinine levels indicative of CKD stage 5 upon admission (see below definition of CKD), or patients on hemodialysis.

Electronic health records (EHR) were used for data extraction. Data included patient demographics, co-morbidities, laboratory tests, prescribed glucose-lowering medications, reason for admission and length of hospitalization. Liver damage was defined as having a diagnosis of cirrhosis (ICD 9 code 571 and sub-codes) or having at least one abnormal laboratory result upon admission: ALT > 52 IU/L, AST > 48 IU/L or ALP > 115 IU/L. 

*Determination of Creatinine change and timing:* All creatinine levels measured during the hospitalization were extracted, and changes in serum creatinine from admission were calculated. Since “baseline creatinine” could not be obtained, patients were marked as having significant change in creatinine (SCIC) if serum creatinine levels rose or dropped ≥ 0.3 mg/dL from admission values at any time during their hospitalization. The premise of this approach was that patients admitted with higher than baseline creatinine levels may show reduction in creatinine levels throughout the hospitalization that may represent recuperation of kidney function. Patients with raising SCIC were termed “deteriorating” and patients with reduction in SCIC were termed “improving”. 

Timing of SCIC was determined based on the first change documented in creatinine levels. If increased creatinine from admission levels were observed, the first day the creatinine increased by ≥0.3 mg/dL was marked as the day of SCIC (i.e., day 1 of hospitalization or later). If decrease in creatinine levels from admission was documented, day of SCIC was the day of hospitalization (i.e., day 0). 

*Severity of SCIC*: Patients were categorized into quintiles of changes from admission creatinine levels. For patients with increase in creatinine levels from admission, the values were calculated as maximum creatinine levels documented minus the minimum creatinine documented. For patients with decrease in creatinine levels from admission, the values were calculated as admission creatinine levels minus the minimum creatinine documented. 

*Determination of eGFR and CKD staging*: eGFR was estimated using the Chronic Kidney Disease Epidemiology Collaboration (CKD-EPI) equation [13]. The values of creatinine used were the ones recorded upon admission. Patients were categorized into CKD stages according to eGFR calculated. 

*Definition and timing of hypoglycemia:* Patients were considered hypoglycemic if they had at least one documented event of hypoglycemia during the hospitalization. An event of hypoglycemia was defined as serum glucose level ≤ 70 mg/dL (3.89 mmol/L)*,* regardless of symptoms, treatment, or method of measurement (chemistry panel or POC (point-of-care)). A distinction was made as to whether the event was recorded by routine blood tests (chemistry panel) or POC results. Date of first hypoglycemic event was documented as well as the number of the hypoglycemic events and severity of the worst event. Timing from SCIC day was calculated. *Severe hypoglycemia* was defined as any serum glucose measurement equal or below 54 mg/dL (3 mmol/L) either by chemistry panel or POC. Average serum glucose levels were calculated by summing all glucose values (chemistry and POC) divided by the number of measurements. 

Definition of diabetes mellitus (DM) [7,14]: Patients were considered as having DM if glucose-lowering medications were recorded upon admission and/or presence of DM as a diagnosis in the discharge letter (ICD9 code 250 and subcodes), and/or hemoglobin A1c above 6.5%. 

*Data analysis*: Data were analyzed using SPSS v. 25.0 (SPSS Inc., Chicago, IL, USA). Continuous variables were compared between patient groups using the t-test for independent samples, or ANOVA with post hoc analysis as appropriate for variable distribution. Associations between nominal variables were assessed using the chi-square test [7]. All continuous variables were tested for co-linearity using multiple linear regressions, and variance inflation factor (VIF) was calculated for all variables. A *p*-value less than 0.05 was considered statistically significant. 

Multiple logistic regression models (backward LR method) were used to examine the association between SCIC status and, separately, SCIC magnitude and incidence of hypoglycemia. For all models, the dependent variable was hypoglycemia, and the independent covariates included sex and age, parameters documented previously to be associated with incident hypoglycemia (i.e., length of stay, admission levels of white blood count, hemoglobin, total cholesterol and albumin, average glucose level during hospitalization, eGFR, acute infection status, liver disease status and diabetes mellitus status) [6,7,8,15,16,17] and SCIC status. A separate model was used to study the effect of SCIC magnitude on hypoglycemia. For this model, with only patients that showed SCIC were included, and the difference between admission and highest or lowest creatinine documented (SCIC magnitude) was used as an independent covariate. Similar models were used for hypoglycemia documented in the laboratory panel. 

Linear regression was used to examine the association between the number of hypoglycemic events with both SCIC status (yes/no) and magnitude. The first model included the number of hypoglycemic events as the dependent variable, and all variables used in the logistic regression model as independent covariates, including SCIC status. A separate model was used for SCIC magnitude.

Cox regression analysis was used to ascertain the association between SCIC and hypoglycemia incidence. The time variable in the model was the day of hypoglycemia incidence from admission (or length of stay for patients without hypoglycemia), and the independent variable included variables used in the logistic regression model (excluding length of hospital stay) and SCIC status (dichotomous) and death during hospitalization as additional covariates. 

## 3. Results

A total of 25,400 patients were included in the study analysis. Mean age of the patients was 69.9 ± 18.0 and 12,524 were males (49.3%). Number of patients defined as having SCIC was 5639 (22.2%); 62.2% of the cases had day of SCIC equal to 0, and 92.7% of patients had day of SCIC lower than 7 days. Table 1 describes patients’ demographic characteristics, comorbidities and baseline laboratory data by SCIC status. Patients with SCIC were older, had longer hospitalization and had more co-morbidities. They had more acute infection as reason for admission. Regarding laboratory tests, patients with SCIC had higher CRP and white blood cells levels, and lower albumin levels.

### 3.1. Association between SCIC Status and Hypoglycemia

Number of patients with hypoglycemia was 1548 (6.1% of patient population). Patients with SCIC had higher incidence of hypoglycemia compared to patients without. This was true for patients with DM (14.9% vs. 5.8%, respectively, *p* < 0.001) and without DM (11.8% vs. 3.4%, respectively, *p* < 0.001). Patients with SCIC had also higher incidence of hypoglycemia documented in chemistry panel among patients with DM (9.9% vs. 3.0%, respectively, *p* < 0.001) and without DM (10.7% vs. 3.1%, respectively, *p*< 0.001).

Logistic regression models showed that SCIC (yes/no) was associated with an increased risk of hypoglycemia (OR 1.853, 95% CI 1.586–2.166, *p* < 0.001). Other covariates associated with increased risk included DM (OR 2.495, 95% CI 2.150–2.895, *p* < 0.001) and length of hospital stay (OR 1.036, 95% CI 1.031–1.042, *p* < 0.001). Factors associated with decreased incidence of hypoglycemia were admission levels of albumin (OR 0.360, 95% CI 0.320–0.406, *p* < 0.001), hemoglobin (OR 0.951, 95% CI 0.923–0.978, *p* = 0.001) and eGFR (OR 0.993, 95% CI 0.990–0.997, *p* < 0.001). Age (OR 0.983, 95% CI 0.979–0.987, *p* < 0.001) and average glucose level (OR 0.990, 95% CI 0.988–0.991, *p* < 0.001) were also associated with decreased risk. Similar results were obtained when analyzing only hypoglycemia events documented in the chemistry panel. The results were similar after omitting the patients that had “improved” SCIC.

**Figure 1:**
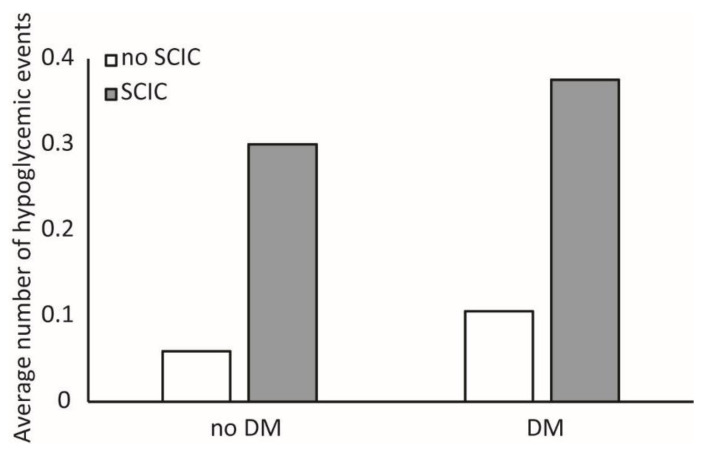
Estimated marginal means of the number of hypoglycemic events according to DM status and significant change in creatinine (SCIC) status.

Among patients with at least one hypoglycemia event, the average number of events recorded during the hospitalization was higher among patients with SCIC (2.5 ± 3.0 vs. 1.8 ± 1.6, *p* < 0.001). To control for longer length of stay and hospital mortality, a linear regression analysis was performed, with the number of events as the dependent variable. The independent covariates were the same as the logistic regression model but also included death during hospitalization (yes/no). The model showed that SCIC was associated with an increased risk for multiple events (β 0.102, 95% CI 0.076–0.128, *p* < 0.001). Additional parameters associated with increased number of hypoglycemic events included length of hospital stay (β 0.015, 95% CI 0.014–0.015, *p* < 0.001), diabetes mellitus status (β 0.144, 95% CI 0.118–0.169, *p* < 0.001) and death during the hospitalization (β 0.243, 95% CI 0.192–0.293, *p* < 0.001). Parameters associated with reduced number of events were older age (β −0.002, 95% CI −0.003–−0.001, *p* < 0.001), higher admission hemoglobin level (β −0.009, 95% CI −0.014–−0.004, *p* = 0.001), higher admission serum albumin (β −0.125, 95% CI −0.146–−0.103, *p* < 0.001), higher average glucose during hospitalization (β −0.002, 95% CI −0.002–−0.001, *p* < 0.001) and acute infection as reason for admission (β −0.033, 95% CI −0.057–−0.009, *p* = 0.009). Similar results were obtained when using only events recorded in the chemistry panel. Figure 1 shows the estimated marginal means of the number of hypoglycemic events according to SCIC status and DM status.

### 3.2. Association between Hypoglycemia and SCIC Magnitude

**Figure 2:**
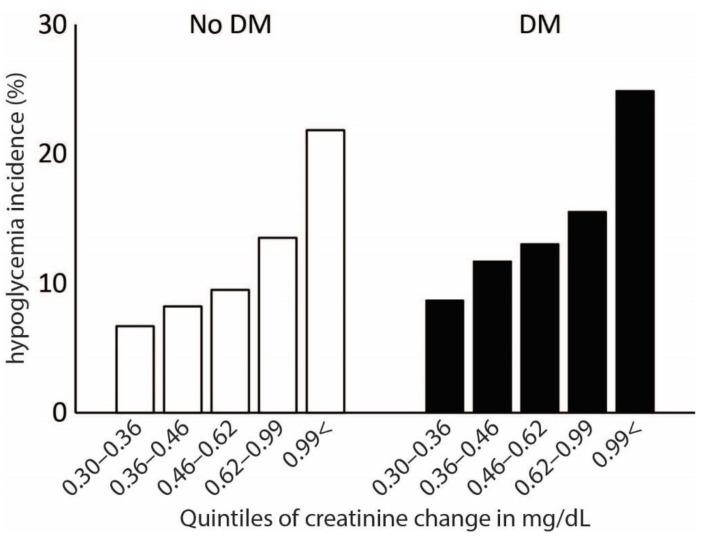
Rate of hypoglycemia incidence across quintiles of creatinine change among patients with significant change in creatinine (SCIC).

Among patients with SCIC, magnitude of creatinine change was significantly associated with hypoglycemia (OR 1.316, 95% CI 1.197–1.447, *p* < 0.001). The model also showed that LOS (OR 1.025, 95% CI 1.020–1.031, *p* < 0.001) and diabetes mellitus status (OR 2.236, 95% CI 1.826–2.737, *p* < 0.001) were associate with increased incidence of hypoglycemia. Older age (OR 0.991, 95% CI 0.985–0.996, *p* = 0.001), higher admission hemoglobin (OR 0.949, 95% CI 0.913–0.987, *p* = 0.008), higher admission serum albumin (OR 0.418, 95% CI 0.359–0.487, *p* < 0.001) and higher average glucose during the hospitalization (OR 0.992, 95% CI 0.990–0.994, *p* < 0.001) were all associated with reduced incidence of hypoglycemia. The rate of hypoglycemia incidence across quintiles of creatinine change among patients with SCIC is described in Figure 2.

Number of hypoglycemia events was also associated with the magnitude of SCIC. The model showed that SCIC was associated with an increased risk for multiple events (β 0.102, 95% CI 0.076–0.128, *p* < 0.001). Additional parameters associated with increased number of hypoglycemic events included length of hospital stay (β 0.015, 95% CI 0.014–0.015, *p* < 0.001), diabetes mellitus status (β 0.144, 95% CI 0.118–0.169, *p* < 0.001) and death during the hospitalization (β 0.243, 95% CI 0.192–0.293, *p* < 0.001). Parameters associated with reduced number of events were older age (β −0.002, 95% CI −0.003–−0.001, *p* < 0.001), higher admission hemoglobin level (β −0.009, 95% CI −0.014–−0.004, *p* = 0.001), higher admission serum albumin (β −0.125, 95% CI −0.146–−0.103, *p* < 0.001), higher average glucose during hospitalization (β −0.002, 95% CI −0.002–−0.001, *p* < 0.001) and acute infection as reason for admission (β −0.033, 95% CI −0.057–−0.009, *p* = 0.009). Again, similar results were obtained when analyzing hypoglycemia events documented in the chemistry panel alone. 

### 3.3. Ascertaining Timing of SCIC and Hypoglycemia

**Figure 3:**
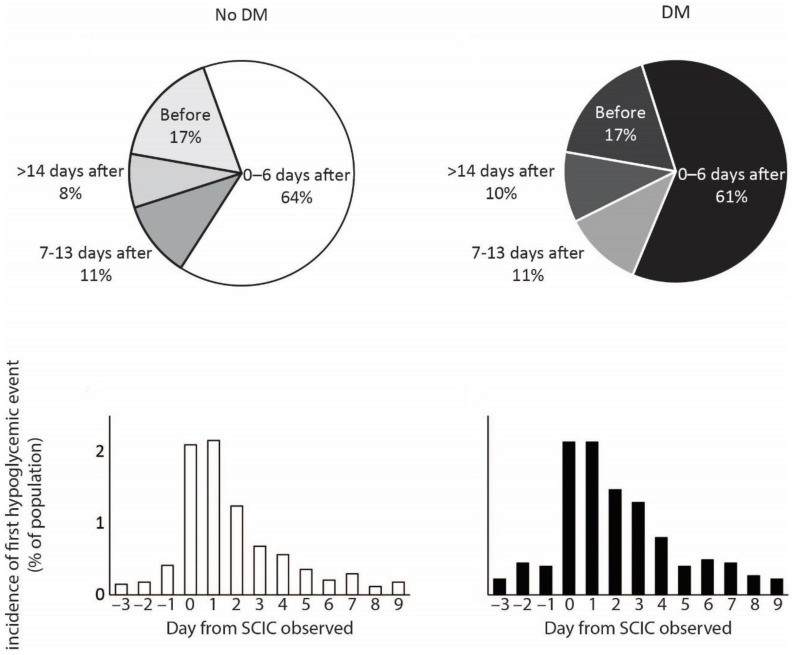
Percent of patients with hypoglycemia according to timing of first hypoglycemic event from significant change in creatinine (SCIC) diagnosis, among patients with (right panels) and without DM (left panels).

Figure 3 shows the percent of patients with hypoglycemia according to timing of first hypoglycemic event from SCIC, among patients with (right panels) and without DM (left panels). More than 60% of patients with hypoglycemia had their first event documented during days 0–6 after SCIC was observed (top panels). This was true for patients with (right panels) and without DM (left panels); 2.1 percent of the hospitalized patients without DM and 2.1% of patients with DM had their first documented hypoglycemic event on day 0 from SCIC. Similar rates were observed on day 1 from SCIC, and this rate showed a gradual decrease throughout the first 5 days from SCIC. Rate of first documented hypoglycemia was maintained relatively low and with similar rates prior to SCIC, and from day 6 onward following SCIC.

**Figure 4:**
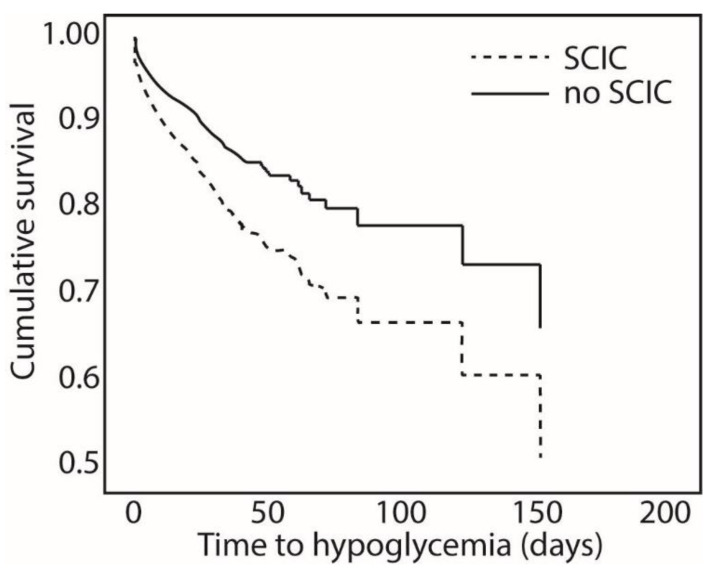
Cumulative survival of patients across time to hypoglycemia and significant change in creatinine (SCIC).

To ascertain the association between SCIC and incident hypoglycemia, a Cox regression analysis was used. The model showed that SCIC was a significant covariate to affect day of first documented hypoglycemia (HR 1.626, 95% CI 1.443–1.831, *p* < 0.001) (Figure 4). Additional covariates to significantly affect the timing of hypoglycemia included average glucose during the hospitalization (HR 0.990, 95% CI 0.989–0.992, *p* < 0.001), diabetes mellitus status (HR 2.420, 95% CI 2.135–2.744, *p* < 0.001), hospital mortality status (HR 1.873, 95% CI 1.600–2.191, *p* < 0.001), acute infection as reason for admission (HR 0.889, 95% CI 0.791–0.999, *p* = 0.048), admission serum albumin (HR 0.499, 95% CI 0.451–0.553, *p* < 0.001) and age (HR 0.987, 95% CI 0.984–0.990, *p* < 0.001). Other covariates such as sex, admission cholesterol and WBC did not affect the timing of hypoglycemia. 

### 3.4. Association between Age, Incident Hypoglycemia and SCIC

The results of our models showed that age was a protective parameter against hypoglycemia, both in incidence and number of events. Given this, we performed several analyses to study the association between age, hypoglycemia and SCIC in our patient population. In a regression model including incident hypoglycemia as the dependent variable and age, sex and diabetes mellitus status as the independent variables, age was positively correlated with incident hypoglycemia (OR 1.004, 95% CI 1.001–1.007, *p* = 0.011). After controlling for SCIC status and eGFR, age was found to be protective against incident hypoglycemia (OR 0.993, 95% CI 0.990–0.997, *p* = 0.001). 

Association between age and SCIC was also investigated. In a regression model using SCIC as the dependent variable, and age as the independent variable, age was associated with increased risk of SCIC (OR 1.029, 95% CI 1.027–1.031, *p* < 0.001). When controlling for admission eGFR, DM status, acute infection as reason for admission, and HTN and CHF as comorbidities, age was associated with lower incidence of SCIC (OR 0.974, 95% CI 0.971–0.977, *p* < 0.001). 

### 3.5. “Deteriorating” SCIC vs. “Improving” SCIC

Table 2 shows the comparison of patients according to positive or negative SCIC. Patients with deteriorated SCIC (i.e., increased serum creatinine during hospitalization) had a longer length of stay; higher rates of CHF, HTN and DM; and higher rates of hypoglycemia during hospitalization.

## 4. Discussion

In the current study, we show that significant changes in serum creatinine levels cause an increase in the incidence of hypoglycemia among non-critically ill in-patients admitted to internal medicine units. This effect was observed among patients with and without DM. More than 60% of hypoglycemic cases occurred during the first week following SCIC, and the rate showed a gradual decline from day 1 after SCIC onward, until a relatively steady rate was observed on day 6 onward. This was again irrespective of DM status and therefore irrespective of glucose-lowering medications. 

The occurrence of SCIC increased the risk of incident hypoglycemia by more than 80% and the number of events by 10%. We also show here that the magnitude of SCIC, presented here as a change from admission creatinine level, was an independent predictor of incident hypoglycemia. Every 0.1 mg/dL change in creatinine accounted for a 31% increase in the risk of hypoglycemia and 5% increase in number of events. While DM status increased the likelihood of hypoglycemia during hospitalization, the effect of SCIC on hypoglycemia was observed among patients with and without DM, to a similar degree. 

These results yet again prove the significant role the kidneys play in the metabolism of glucose. The kidneys maintain serum glucose homeostasis by three different mechanisms: gluconeogenesis, direct uptake from circulation, and glucose reabsorption from the glomerular filtrate [18]. Renal gluconeogenesis is of a great importance for the carbohydrate metabolism, in both physiological and pathological processes [19,20], and has a crucial role in glucose homeostasis [5]. It has been recently shown that gluconeogenesis is impaired in proximal tubule cells among patients with AKI [21], which, in turn, can possibly lead to hypoglycemia. Other kidney mechanism which may be responsible for hypoglycemia is a reduced glucose reabsorption from the glomerular filtrate. Sodium-glucose cotransporter 2 (SGLT2) inhibitors work by limiting glucose reabsorption in the kidney; however, they can inhibit the reabsorption of up to 30–50% of the filtered glucose load [22]. Nevertheless, treatment with SGLT2 inhibitors was found to be associated with hypoglycemia [23]. It might be possible that among patients with AKI, the mechanism of renal glucose reabsorption is damaged and, as a result, leads to hypoglycemia.

We also show here that SCIC was not the only predictor of hypoglycemia. One explanation of our findings may involve malnutrition, which can be defined as an imbalance between consumption and expenditure of either energy, protein or any other nutrient that damages body function [15,24]. ‘Kidney disease wasting’ is a term used to describe occurrence of protein-energy wasting (PEW) in CKD or AKI regardless of the cause [25]. The diagnosis of PEW is based on four characteristics: serum chemistry (low albumin, transthyretin or cholesterol), reduced body mass, reduced muscle mass and reduced dietary intake [25,26]. 

We have previously shown that low admission serum albumin level [16] or a decrease in albumin level during hospital stay [7] is associated with an increased risk of hypoglycemia among patients admitted to internal medicine departments, regardless of DM status. We also showed that low cholesterol level on admission was independently associated with hypoglycemia [6]. In another study, malnutrition risk, as measured by the Nutritional risk screening (NRS2002), almost doubled the risk for hypoglycemia during hospitalization [27]. We show here that serum albumin is a significant predictor of hypoglycemia even after controlling for SCIC and baseline CKD. This may support the idea that the nutritional status of the patient is a predictor of hypoglycemia irrespective of and independently from kidney function.

It should be noted that, among patients with AKI, up to 47% of cases are associated with sepsis (i.e., sepsis associated acute kidney injury) [28]. Sepsis is a major risk factor of incident hypoglycemia by a mechanism not yet elucidated [29,30]. However, in our study, acute infection was not associated with increased incidence of hypoglycemia. Moreover, acute infection was associated with delayed incidence of hypoglycemia, after controlling for day of SCIC. This may suggest that sepsis-induced hypoglycemia is attributed to kidney damage. 

Another finding of our study was the negative association between age and incidence of hypoglycemia. Age, as well as DM disease duration, were found to be associated with hypoglycemia in previous studies [31,32]. While the pathogenesis for this association is not known, it is speculated that this is a result of a defect in the hormonal counterregulatory response, along with a possible failure to recognize the impending hypoglycemia event. In a previous study, we showed that age was associated with hypoglycemia only among patients with eGFR higher than 30 mL/min/1.73 m^2^ [17]. Here, we show that age is associated with hypoglycemia only in univariate analysis, and this association is negated after controlling for renal function and SCIC. It is therefore likely that, the association between age and increased incidence of hypoglycemia is attributed to deteriorated renal function and that after controlling for renal function, disease severity and nutritional status, age does not play a role in incidence of hypoglycemia. This issue needs to be further investigated.

Our study is not without limitations. First and foremost, despite significant efforts to prove a causal relationship between creatinine changes and incident hypoglycemia, we can only suggest such an association. We cannot account for additional factors that may influence the incidence of hypoglycemia, such as fasting on day of admission, taking or withholding medications on day of admission, etc. However, we argue our results are valid since we showed similar results when analyzing cases of SCIC that occurred later than the admission day. 

Furthermore, we cannot conclude that our study population had AKI, given the fact that we did not have a baseline value of creatinine prior to the hospitalization, nor could we analyze urine output. This is why we were able to recognize only significant change in creatinine levels from admission values and not adhere to the AKI definition criteria. However, even after omitting the patients with a reduction in creatinine levels from baseline, the findings were similar in magnitude and significance, meaning that it is possible that the majority of our patients had AKI. Additionally, the definition of SCIC was regardless of baseline creatinine. This implies that the magnitude of creatinine change was not necessarily correlated with the magnitude in reduction of eGFR from baseline values; however, most of the study population had admission serum creatinine values below 2 mg/dL.

## 5. Conclusions

In conclusion, we showed here that significant changes in creatinine levels may be associated with hypoglycemia among patients admitted to internal medicine departments. The association was shown for patients with and without DM. We recommend careful follow up of patients that exhibit any significant change in creatinine levels during their hospitalization, including chemistry tests, nutritional screening and identification of additional risk factors for hypoglycemia.

## Figures and Tables

**Table 1:** Demographic, co-morbidities and admission laboratory data across AKI sub-groups.

Parameter	SCIC n = 5639	no SCIC n = 19,761	*p* Value
Male sex (%)	52.4	48.4	*p* < 0.001
Age (years)	76.1 ± 15.7	68.2 ± 18.3	*p* < 0.001
Acute conditions in discharge letter			
Exacerbation of CHF (%)	9.7	4.9	*p* < 0.001
Exacerbation of COPD (%)	4.6	3.2	*p* < 0.001
Acute coronary syndrome (%)	3.4	1.9	*p* < 0.001
Acute infection (any site) (%)	40.1	19.8	*p* < 0.001
Co-morbidities			
CHF (%)	16.0	8.5	*p* < 0.001
COPD (%)	13.3	10.5	*p* < 0.001
CVA (%)	9.5	7.1	*p* < 0.001
Dementia (%)	8.2	3.9	*p* < 0.001
Hypertension (%)	57.8	50.4	*p* < 0.001
IHD (%)	22.0	18.8	*p* < 0.001
Hyperlipidemia (%)	36.0	35.4	0.384
Liver disease (%)	4.2	3.6	0.029
Malignancy-all types all stages	8.5	6.0	*p* < 0.001
PVD (%)	4.0	2.5	*p* < 0.001
Hypothyroidism (%)	8.4	7.5	*p* = 0.029
Diabetes mellitus (%)	39.9	30.0	*p* < 0.001
Admission measurements and laboratory data			
Temperature (°C)	36.8 ± 0.6	36.8 ± 0.4	*p* < 0.001
Heart rate (BPM)	84.7 ± 18.8	79.8 ± 17.4	*p* < 0.001
Systolic blood pressure (mmHg)	135 ± 28	141 ± 26	*p* < 0.001
Diastolic blood pressure (mmHg)	73 ± 14	78 ± 13	*p* < 0.001
White blood cell count (cells per mm^3^ × 10^3^)	12.7 ± 2.0	10.0 ± 6.4	*p* < 0.001
Hemoglobin (g/dL)	12.2 ± 2.3	12.8 ± 2.0	*p* < 0.001
Sodium (meq/L)	137 ± 6	137 ± 5	*p* < 0.001
Potassium (meq/L)	4.3 ± 0.7	4.1 ± 0.5	*p* < 0.001
Cholesterol (mg/dL)	147 ± 47	165 ± 45	*p* < 0.001
Serum albumin (g/dL)	3.4 ± 0.6	3.9 ± 0.5	*p* < 0.001
C-Reactive Protein (mg/dL)	9.5 ± 10.6	4.1 ± 6.6	*p* < 0.001
Mean glucose during hospitalization (mg/dL)	150 ± 54	133 ± 46	*p* < 0.001
Incident hypoglycemia (%)	13.1	4.1	*p* < 0.001

**Table 2:** Comparison of parameters according to improving/deteriorating SCIC status.

	Deteriorating SCIC n = 2129	Improving SCIC n = 3510	*p* Value
Age (years)	78.7 ± 0.3	74.5 ± 16.6	<0.001
Male sex (%)	49.4	54.2	<0.001
Length of stay (days)	14.5 ± 0.8	8.8 ± 9.3	<0.001
Admission measurements and laboratory data			
Temperature (°C)	36.8 ± 0	36.9 ± 0.5	<0.001
Heart rate (BPM)	86.2 ± 0.4	83.8 ± 18.3	<0.001
Systolic blood pressure (mmHg)	137.3 ± 0.7	133 ± 26.4	<0.001
Diastolic blood pressure (mmHg)	73.6 ± 0.4	72.4 ± 13.4	0.005
White blood cell count (cells per cmm × 10^3^)	12.4 ± 0.2	12.9 ± 10.1	0.041
Hemoglobin (g/dL)	11.8 ± 0	12.4 ± 2.3	<0.001
Cholesterol (mg/dL)	148.2 ± 1.1	146.4 ± 46.3	0.170
Serum albumin (g/dL)	3.4 ± 0	3.5 ± 0.6	<0.001
C-Reactive Protein (mg/dL)	9.2 ± 0.2	9.8 ± 10.8	0.048
Admission creatinine (mg/dL)	1.39 ± 0.65	1.55 ± 0.59	<0.001
Co-morbidities			
Acute infection (any site) (%)	41.2	39.4	0.183
CHF (%)	25.0	10.6	<0.001
Hypertension (%)	62.4	55.0	<0.001
Diabetes mellitus (%)	41.7	38.9	0.037
Incident Hypoglycemia (%)	16.9	10.7	<0.001

## Data Availability

The data that support the findings of this study are available on request from the corresponding author, EL. The data are not publicly available due to privacy or ethical restrictions.
